# Association between perivascular diffusion and white matter microstructural integrity, free water, Aβ burden, and cognition: diffusion tensor vs. kurtosis tensor

**DOI:** 10.3389/fnagi.2026.1733820

**Published:** 2026-03-06

**Authors:** Zhiming Zeng, Xin Jia, Shushu Han, Cuidie Zeng, Jing Bi, Lingchen Liu, Yueming Wu, Tengao Gao, Lei Liang, Fangxiao Cheng

**Affiliations:** 1 Institute of Medical Technology, Peking University Health Science Center, Beijing, China; 2 Beijing Key Laboratory of Magnetic Resonance Imaging Technology, Beijing, China; 3 Department of Nutrition, Fuwai Shenzhen Hospital, Chinese Academy of Medical Sciences, Shenzhen, China; 4 Department of Ultrasound, Aerospace Center Hospital, Beijing, China

**Keywords:** cognitive impairment, diffusion along the perivascular space, free water, peak width of skeletonized mean diffusivity, trajectory patterns

## Abstract

**Background:**

Perivascular diffusion holds great potential for the non-invasive assessment of the glymphatic system (GS). However, Gaussian model-based diffusion tensor imaging analysis along the perivascular space (DTI-ALPS) is limited by microstructural alterations. This study aimed to compare cross-sectional and longitudinal trajectories of diffusion kurtosis imaging ALPS (DKI-ALPS) and DTI-ALPS and investigate their association with white matter (WM) microstructural integrity, free water (FW), Aβ burden, and cognitive impairment (CI).

**Methods:**

This study included 128 healthy controls (HCs) and 83 individuals with cognitive impairment (CI) who underwent multi-shell diffusion-weighted magnetic resonance imaging (dMRI). Four dMRI indices were quantified: DTI-ALPS and DKI-ALPS to assess the GS function; peak width of skeletonized mean diffusivity (PSMD) to evaluate the WM microstructural integrity; and FW-WM to quantify the extracellular fluid accumulation in WM. Cohen’s *d* was reported as the measure of effect size, with generalized linear models (GLMs) adjusting for confounding factors. Functional principal component analysis (FPCA) was used to determine the trajectories of dMRI indices.

**Results:**

CIs exhibited significantly lower DTI-ALPS (1.28 vs. 1.37; *p* = 0.007; Cohen’s *d* = 0.383) and DKI-ALPS (1.37 vs. 1.63, *p* < 0.001; Cohen’s *d* = 0.770) than HCs. GLMs confirmed significant group differences in DKI-ALPS indices. DTI-ALPS was positively correlated with DKI-ALPS (*r* = 0.551; *p* < 0.001), with stronger associations in HCs than in those with CIs (*r* = 0.628 vs. 0.370; all *p* < 0.05). Both DTI-ALPS and DKI-ALPS were negatively correlated with PSMD (*r =* −0.327 and −0.251; all *p* < 0.05) and FW-WM (*r* = −0.317 and −0.393; all *p* < 0.05). The FPCA revealed distinct trajectories of DTI-ALPS, DKI-ALPS, PSMD, and FW-WM between HCs and CIs, and Cohen’s *d* of the first FPC score was 0.685, 0.977, 0.573, and 1.004, respectively (all *p* < 0.001). Compared with baseline dMRI measurements, the trajectory patterns exhibited stronger correlations with Aβ burden (DTI-ALPS, 0.277 vs. −0.217; DKI-ALPS, 0.552 vs. −0.468; PSMD, 0.278 vs. 0.201; FW-WM, 0.313 vs. 0.113) and cognitive performance.

**Conclusion:**

Our study indicated that DKI-ALPS provides an accurate assessment of GS function compared with DTI-ALPS. Longitudinal trajectories, particularly the trajectory of DKI-ALPS, demonstrate stronger associations with Aβ burden and cognitive decline.

## Introduction

Cognitive impairment (CI), primarily associated with aging, is characterized by progressive declines in cognitive and behavioral functions (Langa and Levine, 2014; Jin et al., 2024). Emerging evidence indicates that the accumulation of neurotoxic metabolites (e.g., *β*-amyloid and tau proteins) in brain interstitial fluid contributes to the progressive loss of neuronal structure and function, serving as both a hallmark and driver of the pathogenesis of CI (Rabin et al., 2017; Yang et al., 2023; De Strooper and Karran, 2016). Clearance of these neurotoxic metabolites relies critically on the glymphatic system (GS; Mestre et al., 2020; Iliff et al., 2012). However, assessing GS functions remains challenging: Invasive methods can cause tissue damage, while non-invasive methods require further validation for clinical utility (Keil et al., 2025).

**Figure 1:**
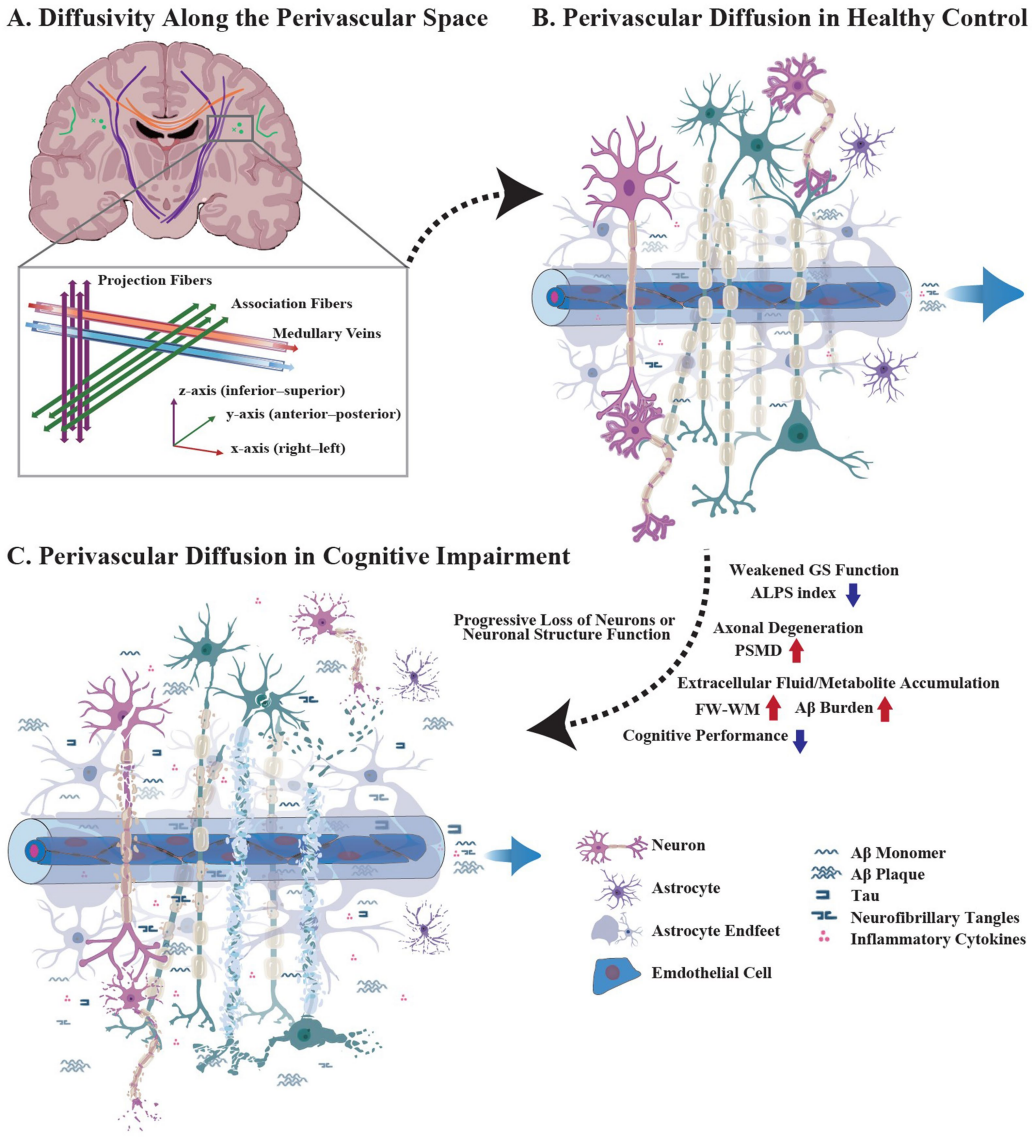
Diffusivity analysis along the perivascular space and the microstructural alterations. **(A)** Diffusivity analysis along the perivascular space. The ALPS index non-invasively evaluates the GS function by differentiating perivascular diffusion from tissue-restricted diffusion. At the level of the lateral ventricles, medullary veins (right–left, *x*-axis) run perpendicular to the ventricular wall, where projection fibers (inferior–superior, *z*-axis) and association fibers (anterior–posterior, *y*-direction) form orthogonal orientations relative to the medullary veins. **(B)** Perivascular diffusion in healthy controls. In healthy states, confounding diffusion signals along the major coordinate axes are frequently observed due to widespread crossing fibers. (**C**) Perivascular diffusion in cognitive impairment. CIs exhibit impaired GS accompanied by a progressive loss of neurons or neuronal structure function. Pathological perivascular microstructural alterations significantly interfere with the differentiation of perivascular diffusion.

The GS facilitates metabolic waste clearance through a directional fluid transport mechanism, which includes the para-arterial influx of cerebrospinal fluid into the brain extracellular spaces through perivascular spaces, followed by the metabolic waste-laden interstitial fluid drained through perivascular efflux pathways of large-caliber veins (Iliff et al., 2012; Rasmussen et al., 2022). Among non-invasive GS assessment methods, diffusivity analysis along the perivascular space (ALPS) based on diffusion tensor imaging (DTI) has become a widely used approach (Taoka et al., 2017; Li et al., 2024). Without requiring exogenous tracers, DTI-ALPS evaluates perivascular activity using second-order diffusion tensors and demonstrates robust correlations with GS function (Iliff et al., 2012). However, Gaussian model-based DTI-ALPS suffers from two critical limitations: (1) fiber crossing—high angular resolution diffusion-weighted magnetic resonance imaging (dMRI) and constrained spherical deconvolution reveal multidirectional fibers in the majority of brain regions, which confound diffusion signals along the coordinate axis (Jeurissen et al., 2013) and (2) radial asymmetry—neurodegenerative diseases exhibit widespread axonal degeneration, violating the radial symmetry assumption fundamental to the decomposition of perivascular diffusion (Wright et al., 2024). Both factors introduce systematic biases in the DTI-ALPS index during perivascular diffusion modeling (Figure 1).

Multi-shell dMRI serves as a powerful tool for probing the microstructural and diffusion properties of neural tissue (Axer and Amunts, 2022; Iima and Le Bihan, 2016). First, multi-shell dMRI enables the quantification of non-Gaussian diffusion properties through diffusion kurtosis imaging (DKI), thereby mitigating biases in DTI-ALPS arising from microstructural alterations (Neto Henriques et al., 2015; Jensen et al., 2005). Second, multi-shell dMRI provides quantitative metrics of white matter (WM) injury through the peak width of skeletonized mean diffusivity (PSMD) and estimates extracellular free water (FW) through bi-tensor models, enabling simultaneous non-invasive assessments of GS function, WM microstructural integrity, and fluid accumulation (Pasternak et al., 2009; Zanon Zotin et al., 2023). The interactions among these processes are critical for elucidating the pathophysiological mechanisms underlying CI; however, their dynamic interactions remain inadequately characterized (Peter et al., 2025).

Therefore, this study aimed to compare the efficacy of DKI and DTI in non-invasively evaluating GS function and to examine their associations with WM microstructural integrity, FW fraction, Aβ burden, and cognitive decline. Additionally, given the highly dynamic nature of the GS, we further investigated the clinical significance of the longitudinal trajectories in tracking neurodegenerative progression.

## Materials and methods

### Participants

The data used in this study were derived from the Alzheimer’s Disease Neuroimaging Initiative (ADNI) database (including ADNI-1, ADNI-GO, ADNI-2, and ADNI-3 studies).^1^ A total of 362 longitudinal imaging sessions were analyzed across 211 participants, comprising healthy controls (HCs) and cognitively impaired individuals (CIs). Detailed inclusion and exclusion criteria are provided in Supplementary method S1. This study is a retrospective study compliant with the Strengthening the Reporting of Observational Studies in Epidemiology (STROBE) guideline (Vandenbroucke et al., 2014).

The neuropsychological assessments were administered using the following tests: the Mini-Mental State Examination (MMSE), Montreal Cognitive Assessment (MoCA), Functional Activities Questionnaire (FAQ), Clinical Dementia Rating Scale Sum of Boxes (CDR-SB), Rey Auditory Verbal Learning Test (RAVLT), Alzheimer’s Disease Assessment Scale-Cognitive Subscale (ADAS-Cog; including ADAS-11, ADAS-13, and ADAS-Q4), Logical Memory Delayed Recall Total Score (LDELTOTAL), and time to complete part B of the Trail Making Test (TRABSCOR). In addition, Aβ burden was evaluated using positron emission tomography (PET) standardized uptake value ratios (SUVRs) of ^18^F-florbetapir (AV45) or ^18^F-florbetaben (FBB), which were calculated following a standardized pipeline (Supplementary method S2).

### MRI acquisition and preprocess

**Figure 2:**
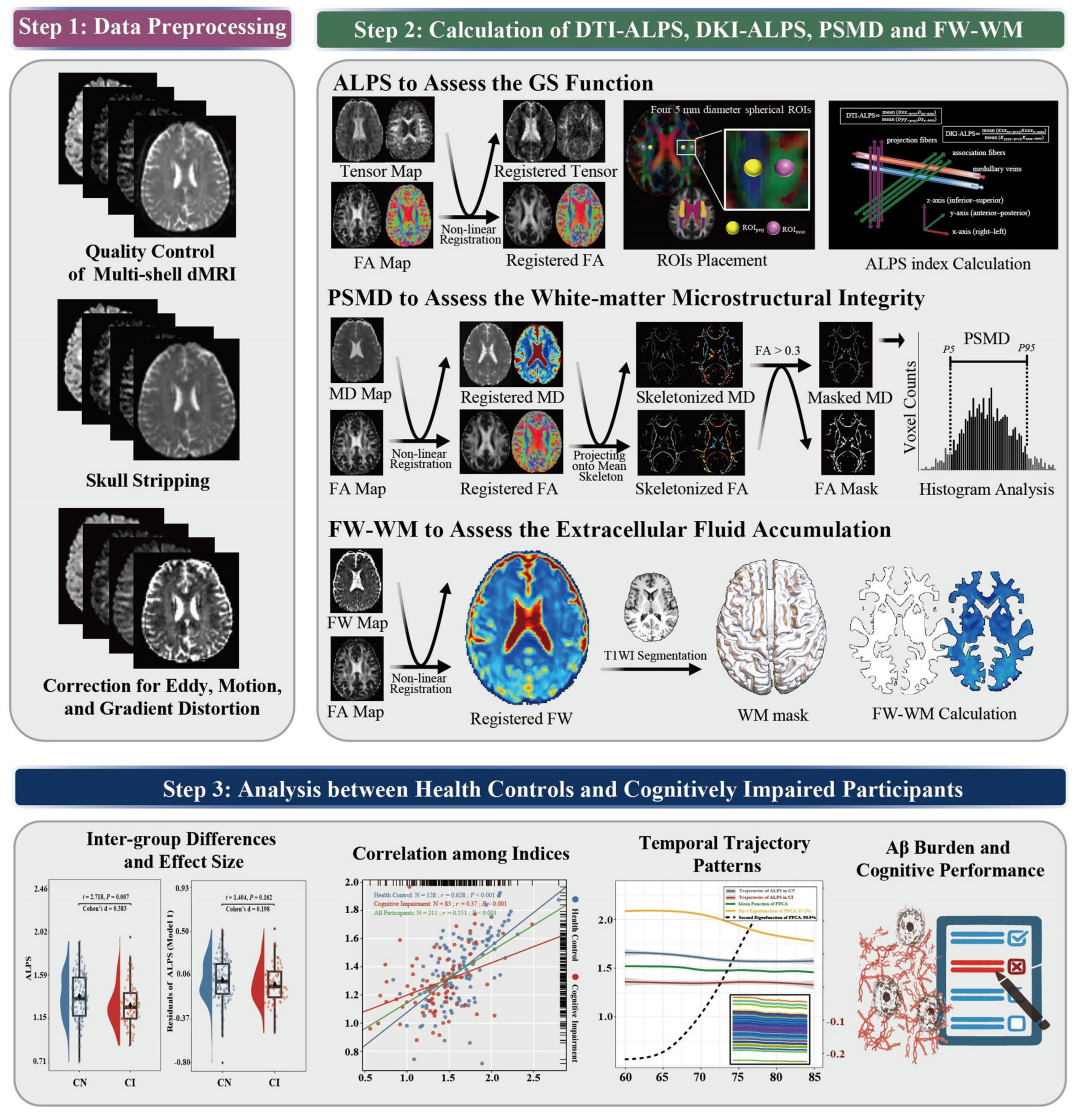
Pipeline for the association between perivascular diffusion and white matter microstructural integrity, free water, Aβ burden, and cognition. Step 1: Data preprocessing included skull stripping, eddy correction, motion correction, and gradient distortion correction; Step 2: Calculation of DTI-ALPS, DKI-ALPS, PSMD, and FW-WM; Step 3: Cross-sectional and longitudinal analyses between HCs and CIs.

dMRI (b values = 0, 500, 1,000, and 2000 s/mm^2^) was acquired for each participant using 3 T scanners with standardized protocols of the ADNI database. More imaging details are provided in Supplementary method S3. Following quality control, dMRI data were preprocessed using a standardized preprocessing pipeline implemented in FSL (FMRIB Software Library, version 6.0.7). Briefly, the preprocessing steps included skull stripping, eddy correction, and motion correction. Gradient distortion correction was subsequently applied to rectify geometric distortions and signal attenuation caused by field inhomogeneity (Figure 2: step 1).

### DTI-ALPS and DKI-ALPS

The ALPS index was calculated from dMRI using a highly reliable pipeline developed and validated by Taoka et al. (2017). Following image preprocessing, fractional anisotropy (FA) maps, as well as diffusion tensor and kurtosis tensor along the x-axis (right–left), y-axis (anterior–posterior), and z-axis (inferior–superior), were extracted. Then, the diffusivity maps were registered to standard space using linear and non-linear transformation matrices generated from the FA map alignment to the MNI152 standard space (FMRIB58 FA template; spatial resolution, 1 × 1 × 1 mm^3^). Four 5-mm diameter spherical regions of interest (ROIs) were initially placed in standard space using fixed coordinates, following the original ALPS methodology. Subsequently, ROI locations were visually inspected in each individual subject and adjusted when necessary to ensure accurate placement within the bilateral projection fibers and association fibers on color-coded FA maps. All ROI placements were independently reviewed by a second investigator, and any discrepancies were resolved by consensus to confirm anatomical validity. We recorded the diffusivity in the directions of the x-axis, y-axis, and z-axis of four ROIs on the projection and association fibers as D_xx_, D_yy_, and D_zz_ for diffusivities of diffusion tensor and K_xxxx_, K_yyyy_, and K_zzzz_ for diffusivities of kurtosis tensor, respectively. The DTI-ALPS and DKI-ALPS indices of the left and right hemispheres were calculated separately, and the ALPS indices of the whole brain were calculated as the average of the bilateral values (Figure 2: step 2).

### Peak width of skeletonized mean diffusivity

PSMD quantified the heterogeneity of mean diffusivity (MD) using a histogram analysis developed by Baykara et al.^2^ and serves as a specific biomarker of WM injury (Baykara et al., 2016). Briefly, the methodological framework combines three main elements: skeletonization, custom mask, and histogram analysis. First, MD and FA maps were registered to the MNI152 standard space using the transformation matrices, and a WM skeleton was generated from the mean FA map thresholded at 0.2. Second, MD maps were projected onto the WM skeleton to obtain MD skeletons, further masked with a mean FA map thresholded at 0.3 to limit partial volume effects with CSF. Finally, PSMD was calculated as the distance between the 5th and 95th percentiles of the MD histogram curve across the WM skeleton (Figure 2: step 2).

### Free water calculation

The bi-tensor models describe the dMRI signal as a weighted mixture of brain tissue (anisotropic tensor) and FW (isotropic tensor with fixed diffusion constant), resulting in an FW-corrected tensor map and an FW map (Pasternak et al., 2009). FW can only be found in the extracellular space and is influenced by microstructural disruption and GS function. Multi-shell dMRI, traditionally considered more accurate for quantifying FW, was used in this study. FW quantification was implemented using the DIPY package in Python,^3^ following established multi-shell processing protocols. Subsequently, the resulting FW map was registered to standard space, and the mean cerebral FW-WM was extracted using the T1WI-based WM mask delineated through FAST (FMRIB’s Automated Segmentation Tool; Figure 2: step 2).

### Longitudinal trajectories analysis

A functional principal component analysis (FPCA) was used to determine different trajectory patterns of DTI-ALPS, DKI-ALPS, PSMD, and FW-WM. The FPCA transforms sparse longitudinal measures with irregular time intervals into continuous functional curves through Karhunen–Loève expansion, decomposed as population mean functions and FPC scores–weighted eigenfunctions. The individual FPC scores represent distinct trajectory patterns. More detailed FPCAs are provided in Supplementary method S4. The number of FPCs was determined using a cumulative explained variance ratio thresholded at 95%.

### Statistical analysis

All statistical analyses were performed using R (version 4.2.0)^4^ and Statistical Package for the Social Sciences (SPSS; version 27.0, IBM Corporation) software. The code is available on GitHub.^5^ The continuous variables were displayed as mean ± standard deviation (SD) and compared using *t*-tests. The categorical variables were displayed as frequencies (percentages) and compared using χ^2^ tests. The Kolmogorov–Smirnov test was used to assess data distribution. Cohen’s *d* was reported as the measure of effect size for between-group comparisons. A two-tailed *p-*value of < 0.05 was considered statistically significant.

To minimize confounding effects, between-group differences in dMRI indices were also evaluated using a general linear model (GLM) while controlling for age, sex, and years of education (Model 1). Furthermore, to determine the influence of PSMD and FW-WM on the measurements of GS function, we also included PSMD and FW-WM as confounding factors in Model 2, which included the covariates of age, sex, years of education, PSMD, and FW-WM, for the evaluation of DTI-ALPS and DKI-ALPS. Pearson’s correlation analyses were used to assess the correlations among DTI-ALPS, DKI-ALPS, PSMD, FW-WM, and Aβ deposition. For non-normally distributed variables of neuropsychological scores, Spearman’s rank correlation tests were applied to evaluate the association between dMRI indices and cognitive performance, followed by false discovery rate (FDR) correction for multiple comparisons.

## Results

### Demographic and cognitive characteristics of the study population

This study enrolled 128 male HCs (46 [35.94%], with a mean age of 71.47 ± 7.30 years) and 83 male CIs (48 [57.83%], with a mean age of 72.21 ± 6.45 years; Table 1). No significant differences in age (*p* = 0.456) and apolipoprotein E (APOEε4) status (*p* = 0.052) were found between HCs and CIs. Compared with HCs, CIs exhibited a significantly higher proportion of male participants and a lower year of education (all *p* < 0.05). CIs also showed significantly higher Aβ burden than HCs (*p* < 0.001).

**Table 1:** Demographic and cognitive characteristics of the study population.

Characteristics	Overall (*N* = 211)	HCs (*N* = 128)	CIs (*N* = 83)	*p*-value	SMD
Age (y)	71.76 ± 6.97	71.47 ± 7.30	72.21 ± 6.45	0.456	0.107
Sex				0.003*	0.450
Male	94 (44.55)	46 (35.94)	48 (57.83)		
Female	117 (55.45)	82 (64.06)	35 (42.17)		
Education (y)	16.22 ± 2.51	16.55 ± 2.42	15.71 ± 2.56	0.018*	0.335
APOEε4				0.052	0.368
No alleles	110 (67.07)	75 (70.09)	35 (61.40)		
One allele	44 (26.83)	29 (27.10)	15 (26.32)		
Two alleles	10 (6.10)	3 (2.80)	7 (12.28)		
Aβ (SUVRs)	1.15 ± 0.22	1.09 ± 0.17	1.25 ± 0.24	<0.001*	0.733
MMSE	28.22 ± 2.79	29.17 ± 0.98	26.73 ± 3.87	<0.001*	0.867
MoCA	24.41 ± 3.94	25.87 ± 2.61	22.06 ± 4.56	<0.001*	1.025
FAQ	1.97 ± 4.75	0.14 ± 0.73	4.77 ± 6.60	<0.001*	0.985
CDR-SB	0.79 ± 1.56	0.04 ± 0.14	1.96 ± 2.00	<0.001*	1.359
RAVLT					
Immediate	42.00 ± 12.58	46.55 ± 11.09	34.81 ± 11.43	<0.001*	1.042
Learning	5.31 ± 2.85	6.09 ± 2.59	4.07 ± 2.81	<0.001*	0.746
Forgetting	4.24 ± 2.85	3.64 ± 2.66	5.19 ± 2.89	<0.001*	0.556
Percent forgetting	47.27 ± 34.19	34.81 ± 27.52	66.97 ± 34.57	<0.001*	1.029
ADAS-Cog					
ADAS-11	7.20 ± 4.93	5.24 ± 2.54	10.26 ± 6.09	<0.001*	1.074
ADAS-13	11.41 ± 7.46	8.24 ± 4.05	16.37 ± 8.80	<0.001*	1.187
ADAS-Q4	3.71 ± 2.60	2.69 ± 1.80	5.33 ± 2.85	<0.001*	1.110
LDELTOTAL	10.79 ± 5.42	13.23 ± 4.05	6.93 ± 5.06	<0.001*	1.377
TRABSCOR	88.30 ± 50.06	74.01 ± 35.30	111.10 ± 60.84	<0.001*	0.746

HC, healthy control; CI, cognitive impairment; SMD, standard mean difference; APOEε4, apolipoprotein E ε4 allele; Aβ, amyloid β; SUVR, standardized uptake value ratio; MMSE, Mini-Mental State Examination; MoCA, Montreal Cognitive Assessment; FAQ, Functional Activities Questionnaire; CDR-SB, Clinical Dementia Rating Scale Sum of Boxes; RAVLT, Rey Auditory Verbal Learning Test; ADAS-Cog, Alzheimer’s Disease Assessment Scale–Cognitive Subscale; LDELTOTAL, Logical Memory Delayed Recall Total Score; TRABSCOR, time to complete part B of the Trail Making Test. Continuous variables are presented as means ± SDs, and groups were compared using the t-test. The categorical variable sex is presented as number of participants with percentages in parentheses, and groups were compared using the χ^2^ test. *p-value was <0.05 and considered statistically significant.

CIs exhibited significantly poorer cognitive performance than HCs in all neuropsychological assessments (all *p* < 0.001). Specifically, CIs scored significantly lower than HCs on the MMSE, MoCA, RAVLT (immediate and learning), and LDELTOTAL. They scored higher on the FAQ, CDR-SB, ADAS-Cog (ADAS-11, ADAS-13, and ADAS-Q4), and RAVLT (forgetting and percent forgetting).

### Between-group differences in dMRI indices

**Figure 3:**
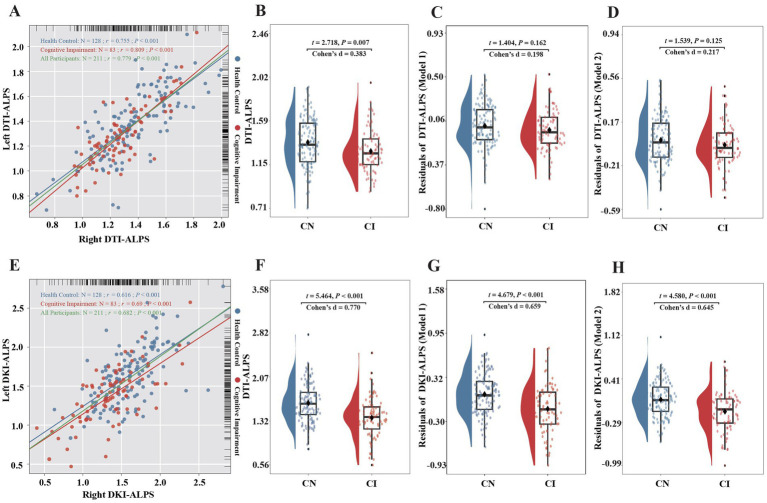
Between-group difference in DTI-ALPS and DKI-ALPS indices. **(A)** Correlation between the left and right DTI-ALPS indices. **(B)** Between-group difference in DTI-ALPS indices. **(C)** Between-group difference in DTI-ALPS indices while controlling for age, sex, and years of education (Model 1). **(D)** Between-group difference in DTI-ALPS indices while controlling for age, sex, years of education, PSMD, and FW-WM (Model 2). **(E)** Correlation between the left and right DKI-ALPS indices. **(F)** Between-group difference in DKI-ALPS indices. **(G)** Between-group difference in DKI-ALPS indices while controlling for age, sex, and years of education (Model 1). **(H)** Between-group difference in DKI-ALPS indices while controlling for age, sex, years of education, PSMD, and FW-WM (Model 2).

Strong positive correlations were observed between the left and right ALPS indices. The correlations between left and right DKI-ALPS (*r =* 0.616, 0.69, and 0.682 for HCs, CIs, and all participants, respectively; all *p* < 0.001) were weaker than those of DTI-ALPS (*r =* 0.755, 0.809, and 0.779 for HCs, CIs, and all participants, respectively; all *p* < 0.001; Figures 3A,E). Compared with HCs, CIs exhibited significantly lower DTI-ALPS (1.28 vs. 1.37; *p* = 0.007) and DKI-ALPS (1.37 vs. 1.63; *p* < 0.001), and DKI-ALPS exhibited a larger effect size than DTI-ALPS (Cohen’s *d*, 0.770 vs. 0.383; Figures 3B,F). CIs also showed marginally higher PSMD (0.25 × 10^−3^ vs. 0.26 × 10^−3^, *p* = 0.777) and FW-WM (0.28 vs. 0.29, *p* = 0.071) than HCs, although these differences were not statistically significant (Supplementary Table S1).

After adjusting for age, sex, and years of education (Model 1), no significant between-group differences were observed for DTI-ALPS (*p* = 0.162; Cohen’s *d* = 0.198), whereas DKI-ALPS indices were significantly lower in CIs than in HCs (*p* < 0.001; Cohen’s *d* = 0.659; Figures 3C,G). Similar results were also observed in the left and right DTI-ALPS and DKI-ALPS indices (Supplementary Table S2). PSMD and FW-WM showed no significant group differences while controlling for age, sex, and years of education (Model 1; all *p* > 0.05). After adjusting for age, sex, years of education, PSMD, and FW-WM (Model 2), no significant between-group differences were observed for DTI-ALPS (*p* = 0.125; Cohen’s *d* = 0.217), whereas significantly lower DKI-ALPS indices were also consistently observed in CIs (*p* < 0.001; Cohen’s *d* = 0.645; Figures 3D,H).

### Correlations among dMRI indices

**Figure 4:**
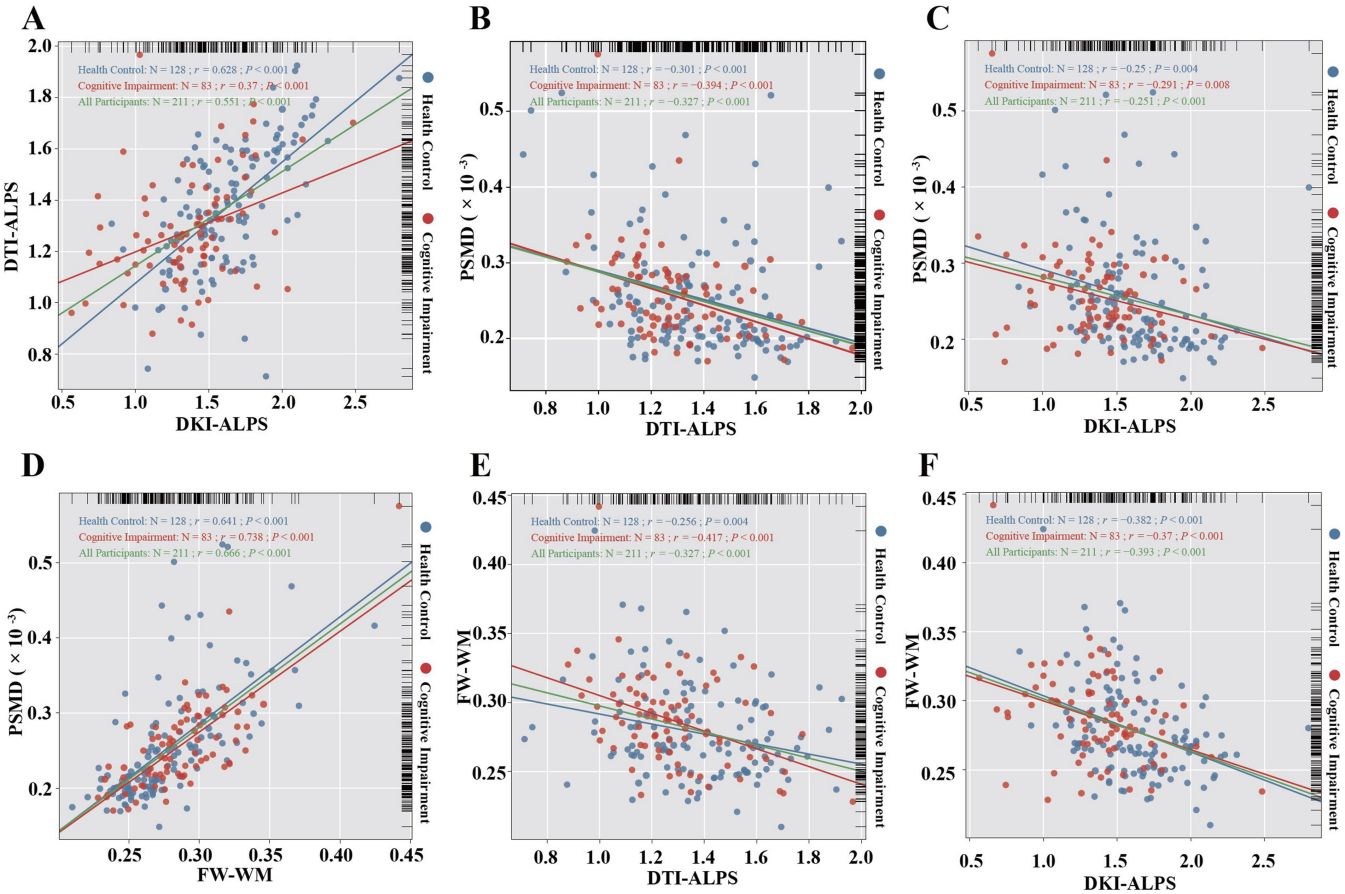
Correlations among DTI-ALPS, DKI-ALPS, PSMD, and FW-WM. **(A)** Correlation between DTI-ALPS and DKI-ALPS. **(B)** Correlation between PSMD and DTI-ALPS. **(C)** Correlation between PSMD and DKI-ALPS. **(D)** Correlation between PSMD and FW-WM. **(E)** Correlation between FW-WM and DTI-ALPS. **(F)** Correlation between FW-WM and DKI-ALPS.

Significant positive correlations were observed between DTI-ALPS and DKI-ALPS (*r =* 0.551; *p* < 0.001), with differential correlation coefficients observed between HCs and CIs (*r =* 0.628 vs. 0.37; all *p* < 0.001; Figure 4A). Similar results were also observed for the left and right DTI-ALPS and DKI-ALPS indices (Supplementary Table S3). The observed between-group differences in correlation coefficients suggest that DTI-ALPS and DKI-ALPS may exhibit differential sensitivity to pathophysiological processes of CI.

PSMD was negatively correlated with ALPS indices, demonstrating stronger associations with DTI-ALPS (*r =* −0.301, −0.394, and −0.327 for HCs, CIs, and all participants, respectively; all *p* < 0.001) than with DKI-ALPS (*r =* −0.25, −0.291, and −0.251 for HCs, CIs, and all participants, respectively; all *p* < 0.001; Figures 4B,C). FW-WM showed robust positive correlations with PSMD (*r =* 0.641, 0.738, and 0.666 for HCs, CIs, and all participants, respectively; all *p* < 0.001; Figure 4D). Notably, FW-WM was negatively correlated with ALPS indices, demonstrating stronger associations with DKI-ALPS (*r = −*0.382, −0.37, and −0.393 for HCs, CIs, and all participants, respectively; all *p* < 0.001) than with DTI-ALPS (*r = −*0.256, −0.417, and −0.327 for HCs, CIs, and all participants, respectively; all *p* < 0.05; Figures 4E,F).

### Longitudinal trajectories in dMRI indices

**Figure 5:**
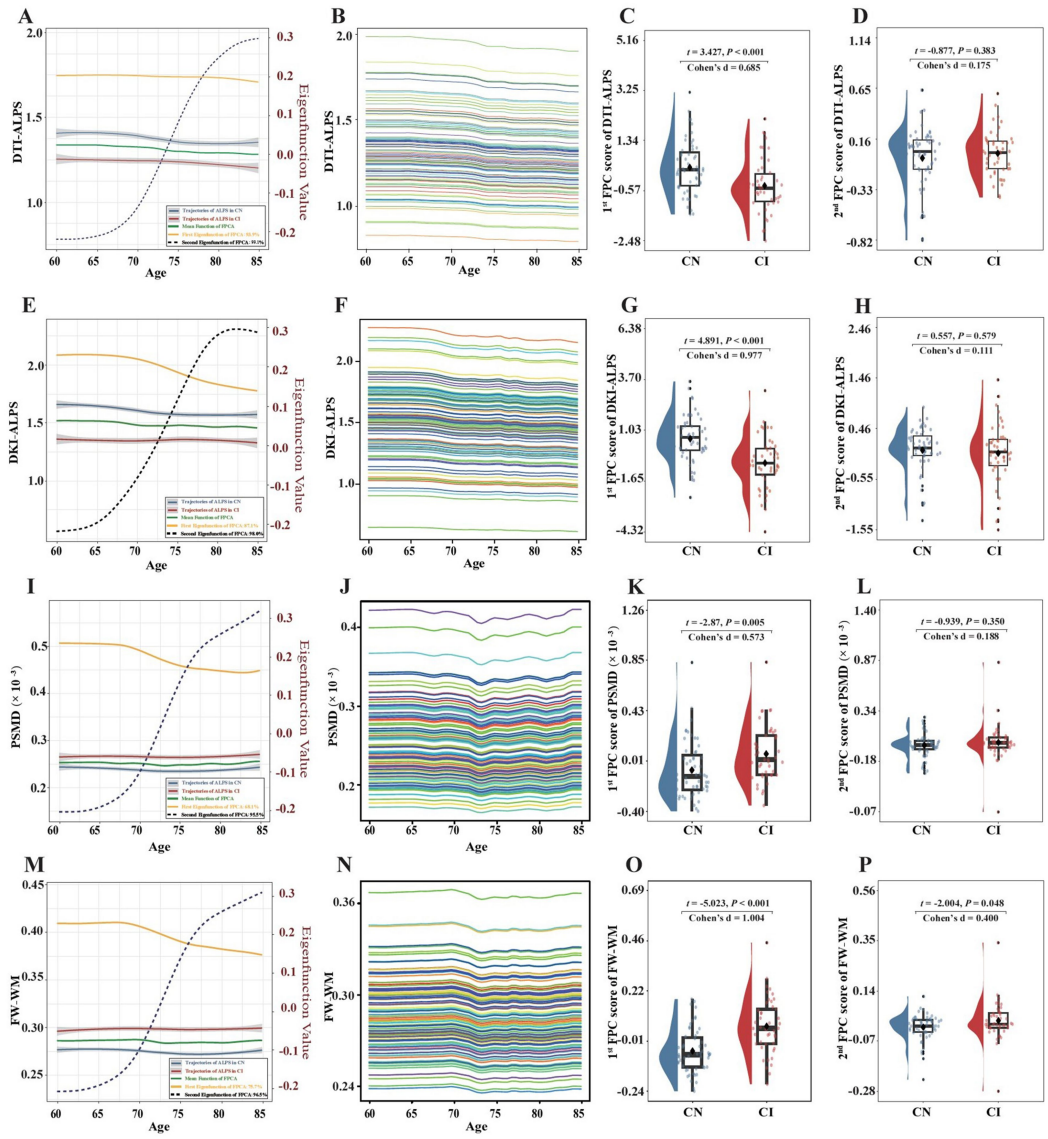
Longitudinal trajectories in DTI-ALPS, DKI-ALPS, PSMD, and FW-WM. **(A)** The mean functions and eigenfunctions of DTI-ALPS. **(B)** The first singular value decomposition component of DTI-ALPS. **(C)** Between-group difference in the first FPC score of DTI-ALPS. **(D)** Between-group difference in the second FPC score of DTI-ALPS. **(E)** The mean functions and eigenfunctions of DKI-ALPS. **(F)** The first singular value decomposition component of DKI-ALPS. **(G)** Between-group difference in the first FPC score of DKI-ALPS. **(H)** Between-group difference in the second FPC score of DKI-ALPS. **(I)** The mean functions and eigenfunctions of PSMD. **(J)** The first singular value decomposition component of PSMD. **(K)** Between-group difference in the first FPC score of PSMD. **(L)** Between-group difference in the second FPC score of PSMD. **(M)** The mean functions and eigenfunctions of FW-WM. **(N)** The first singular value decomposition component of FW-WM. **(O)** Between-group difference in the first FPC score of FW-WM. **(P)** Between-group difference in the second FPC score of FW-WM.

FPCA revealed divergent longitudinal trajectories of DTI-ALPS, DKI-ALPS, PSMD, and FW-WM between HCs and CIs (Figure 5). Compared with HCs, CIs exhibited lower baseline measurements but slower age-related declines in DTI-ALPS and DKI-ALPS. In contrast, CIs exhibited higher baseline measurements with increases in PSMD and FW-WM. The first FPC scores differed significantly between HCs and CIs across all dMRI indices: DTI-ALPS (*p* < 0.001; Cohen’s *d* = 0.685), DKI-ALPS (*p* < 0.001; Cohen’s *d* = 0.977), PSMD (*p* < 0.001; Cohen’s *d* = 0.573), and FW-WM (*p* < 0.001; Cohen’s *d* = 1.004). Only the second FPC score of FW-WM showed a marginal group difference (*p* = 0.048; *d* = 0.4; Supplementary Table S4).

Significant positive correlations were observed between the first FPC scores of DTI-ALPS and DKI-ALPS (*r =* 0.685, 0.455, and 0.629 for HCs, CIs, and all participants, respectively; all *p* < 0.001; Supplementary Table S5). Similarly, the first FPC scores of PSMD and FW-WM showed positive correlations (*r =* 0.596, 0.640, and 0.651 for HCs, CIs, and all participants, respectively; all *p* < 0.001). Unlike baseline measurements, the first FPC score of DKI-ALPS exhibited stronger negative correlations with the first FPC scores of PSMD (*r =* −0.458 vs. −0.318; all *p* < 0.001) and FW-WM (*r =* −0.545 vs. −0.365; all *p* < 0.001) compared with the first FPC score of DTI-ALPS.

### Correlation between dMRI indices and Aβ deposition

Negative correlations with Aβ SUVRs were observed in both DTI-ALPS and DKI-ALPS, with stronger effects in DKI-ALPS (*r =* −0.468 vs. −0.217; all *p* < 0.05; Supplementary Table S6). FW-WM showed a weak correlation with Aβ SUVRs (*r* = 0.201, *p* = 0.011), while no significant correlation was observed between PSMD and Aβ SUVRs (*r* = 0.113, *p* = 0.157). The correlations between Aβ SUVRs and the first FPC scores of DTI-ALPS, DKI-ALPS, PSMD, and FW-WM were 0.277, 0.552, 0.278, and 0.313, respectively (all *p* < 0.05).

### Correlation between dMRI indices and cognitive function

Both DTI-ALPS and DKI-ALPS were negatively correlated with cognitive performance, with DKI-ALPS exhibiting stronger associations than DTI-ALPS across the majority of neuropsychological assessments (Supplementary Table S7). Specifically, lower ALPS indices were associated with worse scores on the MMSE, MoCA, RAVLT (immediate and learning), and LDELTOTAL (all *P_fdr_* < 0.05). Concurrently, lower ALPS indices were associated with higher scores on the FAQ, CDR-SB, RAVLT (forgetting and percent forgetting), ADAS-Cog (ADAS-11, ADAS-13, and ADAS-Q4), and TRABSCOR (all *P_fdr_* < 0.05). Elevated PSMD was correlated with higher CDR-SB scores (*P_fdr_* < 0.05). Elevated FW-WM was associated with better scores on the FAQ, CDR-SB, RAVLT (percent forgetting), ADAS-Cog (ADAS-11, ADAS-13, and ADAS-Q4), TRABSCOR, RAVLT (immediate), and LDELTOTAL (all *P_fdr_* < 0.05). The first FPC scores of DTI-ALPS, DKI-ALPS, PSMD, and FW-WM demonstrated stronger or comparable associations with cognitive decline than baseline dMRI measurements (Supplementary Table S8).

## Discussion

Perivascular diffusion plays a key role in maintaining brain homeostasis through a directional fluid transport mechanism mediated by para-arterial (influx) and para-venous (efflux) perivascular spaces, which are closely associated with pathological protein accumulation (e.g., Aβ) and cognitive decline in CIs (Huang et al., 2024; Li et al., 2024). However, DTI-ALPS is limited by microstructural complexity stemming from fiber crossings and microstructural alterations, introducing systematic biases in the assessment of perivascular diffusion (Wright et al., 2024; Jeurissen et al., 2013). Furthermore, GS function, WH alterations, and FW fraction synergistically drive CI progression, yet the correlations and interactions among them remain inadequately characterized (Peter et al., 2025). In this study, we jointly quantified DTI-ALPS, DKI-ALPS, PSMD, and FW-WM, systematically examining their interrelationships, longitudinal trajectories, and associations with Aβ burden and cognitive performance. We found that (1) CIs exhibited significantly reduced perivascular diffusion compared with HCs. Within this context, DKI-ALPS showed larger effect sizes and stronger associations with Aβ burden and cognitive measures than DTI-ALPS; (2) PSMD exhibited a strong positive association with FW-WM, while DTI-ALPS was more closely associated with PSMD and less strongly associated with FW-WM than DKI-ALPS, suggesting differential sensitivity of ALPS metrics to microstructural integrity and extracellular fluid alterations; and (3) CIs and HCs exhibited divergent longitudinal trajectories across all diffusion-derived indices. Trajectory-based measures showed stronger associations with Aβ burden and cognitive performance than corresponding baseline dMRI measurements.

The ALPS index measures the fluid diffusivity along the perivascular space and is an effective biomarker of GS function (Li et al., 2024; Chen et al., 2025). The anatomical foundation of the ALPS index is rooted in a unique structure at the level of the lateral ventricles, featuring three mutually perpendicular components: medullary veins (x-axis), projection fibers (z-axis), and association fibers (y-axis; Shapiro et al., 2023). This unique spatial arrangement allows the differentiation between perivascular diffusion and tissue-restricted diffusion using the diffusion tensor. In our study, a lower DTI-ALPS index was associated with poorer cognitive performance, which aligns with prior findings of Taoka et al. (2017) and Zhang et al. (2024). However, Gaussian model-based DTI provides a limited interpretation of complex structure (Schilling et al., 2025). DTI-ALPS is limited by systematic biases arising from fiber crossings, axonal damage, and other WM microstructural alterations. DTI-ALPS may primarily reflect local geometric changes rather than the function of GS. The bias introduced by microstructural alterations appears to represent a fundamental limitation of the assumptions of DTI rather than an issue that can be resolved through enhanced spatial resolution or stronger gradients (Weber et al., 2015; Cao et al., 2024; Schilling et al., 2017). DKI addresses this limitation by quantifying non-Gaussian diffusion properties, providing more accurate estimates of microstructural integrity (Cheng et al., 2018; Cao et al., 2024). A significant finding of our study is that DKI-ALPS outperforms DTI-ALPS in detecting group differences and shows stronger correlations with Aβ burden and cognitive decline.

Our study first revealed that ALPS indices were negatively correlated with PSMD and FW-WM, while PSMD and FW-WM showed a robust correlation. These findings corroborate the fundamental hypothesis that GS dysfunction leads to fluid/neurotoxic metabolite accumulation, contributing to increased FW fraction and white matter microstructural damage (Mestre et al., 2020). PSMD reflects WM injury rather than disease-specific pathology, and elevated PSMD is predominantly observed in Alzheimer’s disease patients with significant WM degeneration (Baykara et al., 2016). Histological evidence indicated that the loss of neurons or cellular structures contributes to expanded extracellular spaces, partially accounting for the robust correlation between FW-WM and PSMD (Hoy et al., 2017). Furthermore, the concurrent accumulation of neurotoxic metabolites and FW synergistically drives progressive WM injury (Wang et al., 2025; Mogensen et al., 2021). Given the interactions among perivascular diffusion, WM damage, and FW fraction, causal inferences should be interpreted cautiously (Duering et al., 2018; Pasternak et al., 2016; Peter et al., 2025). Our study revealed a novel and significant finding: PSMD was more strongly correlated with DTI-ALPS than with DKI-ALPS, whereas FW-WM exhibited significantly stronger correlations with DKI-ALPS. The between-group disparities in correlations indicated that DTI-ALPS and DKI-ALPS exhibit differential sensitivity to the neurodegenerative processes of CI: DTI-ALPS predominantly reflects tract-specific WM microstructural integrity, while DKI-ALPS captures perivascular diffusion alterations associated with GS dysfunction and fluid/metabolite accumulation (Taoka et al., 2017; Iliff et al., 2012). This interpretation was further confirmed by the correlation analyses in the HC and CI subgroups.

GS maturation is a protracted process, with its function peaking around middle age (~40 years) and declining progressively thereafter (Taoka et al., 2022; Zhou et al., 2020). Our findings align with prior reports of age-related ALPS reductions in middle-aged and older adults (Sharkey et al., 2024). GS dysfunction, a pivotal pathophysiological event in neurodegenerative diseases, precedes both Aβ deposition and cognitive decline (Peng et al., 2016). Monitoring the dynamics of GS function may provide valuable insights into the progression of neurodegenerative diseases. However, longitudinal analyses predominantly focused on fixed-interval ALPS delta values, neglecting trajectory patterns that could better predict Aβ accumulation and cognitive decline (Yu et al., 2025). Our findings revealed divergent ALPS trajectories between CIs and HCs: HCs exhibited higher baseline ALPS indices but showed steeper age-related declines. Additionally, CIs and HCs exhibited divergent longitudinal trajectories in both PSMD and FW-WM, with the CIs demonstrating higher baseline measurements accompanied by progressive increases in both parameters over time. Longitudinal trajectories of dMRI indices, particularly the trajectory of DKI-ALPS, showed stronger correlations with Aβ burden and cognitive performance than cross-sectional measures. Collectively, these results underscore the significance of trajectory patterns of dMRI measures in tracking early GS dysfunction and the progression of neurodegenerative diseases.

The strengths of this study include its multi-biomarker analysis using multi-shell dMRI and longitudinal trajectory analysis to map dynamic neurodegenerative processes, while also clarifying the systematic biases inherent in DTI-ALPS and identifying the distinct trajectory patterns between CIs and HCs. However, several limitations should be acknowledged. First, the ALPS index provides a region-specific assessment of GS function and cannot serve as an accurate measure of the whole brain due to heterogeneity in vascular density and AQP4 expression. Nevertheless, existing neuroimaging evidence has shown significant concordance between the ALPS index and gadolinium-based contrast agent quantification in evaluating GS function (Zhang et al., 2021). Second, variations in dMRI acquisition parameters, such as voxel size, repetition time, and echo time, may introduce biases in the extraction of dMRI biomarkers, potentially confounding the comparisons between HCs and CIs. Third, region-wise PSMD and FW-WM analyses were not performed; although global measures are robust, regional analyses might provide additional insights into diffusion–glymphatic interactions. Then, a critical limitation stems from the lack of histopathological validation for dMRI biomarkers, as dMRI measurements may not fully capture the underlying pathological processes. Finally, the restricted duration of longitudinal follow-up limited the trajectory analysis, necessitating extended follow-ups for temporal modeling.

## Conclusion

Our study demonstrated that DKI-ALPS provides a more accurate assessment of GS function than DTI-ALPS. CIs and HCs exhibited distinct longitudinal trajectories of DTI-ALPS, DKI-ALPS, PSMD, and FW-WM, with these trajectories, particularly that of DKI-ALPS, showing stronger associations with Aβ burden and cognitive performance than baseline dMRI measurements.

## Data Availability

The datasets presented in this study can be found in online repositories. The names of the repository/repositories and accession number(s) can be found at: https://github.com/ZM50149/dMRI-ALPS.

## Footnotes

1
https://adni.loni.usc.edu/

2
www.psmd-marker.com

3
https://docs.dipy.org/

4
http://www.R-project.org

5
https://github.com/ZM50149/dMRI-ALPS
