# Supplementary material for: Association between perivascular diffusion and white matter microstructural integrity, free water, Aβ burden, and cognition: diffusion tensor vs. kurtosis tensor
Source: Front Aging Neurosci. 2026 Mar 6;18:1733820. doi: 10.3389/fnagi.2026.1733820 (PMC13002833; doi:10.3389/fnagi.2026.1733820)
Supplement: Supplementary file 1 [file Data_Sheet_1.docx]

**Association between perivascular diffusion and white matter microstructural integrity, free water, Aβ burden, and cognition: diffusion tensor vs. kurtosis tensor**

Zhiming Zeng^1,2^, Xin Jia^1,2^, Shushu Han^3^, Cuidie Zeng^1,2^, Jing Bi^1,2^, Lingchen Liu^1,2^, Yueming Wu^1,2^, Tengao Gao^1,2^, Lei Liang^4,^**^*^**, Fangxiao Cheng^1,2,^**^*^**

^1^Institute of Medical Technology, Peking University Health Science Center, Beijing, China.

^2^Beijing Key Laboratory of Magnetic Resonance Imaging Technology, Beijing, China.

^3^Department of Nutrition, Fuwai Shenzhen Hospital, Chinese Academy of Medical Sciences, Shenzhen, China.

^4^Department of Ultrasound, Aerospace Center Hospital, Beijing, China.

**^*^Correspondence to**

Fangxiao Cheng, Institute of Medical Technology, Peking University Health Science Center, 38 Xueyuan Road, Haidian District, Beijing 100191, China (e-mail: chengfangxiao@bjmu.edu.cn);

Lei Liang, Department of Ultrasound, Aerospace Center Hospital, 15 Yuquan Road, Haidian District, Beijing 100049, China (e-mail: [lianglei_csk@126.com](mailto:lianglei_csk@126.com)).

**METHOD S1. Patients Inclusion and Exclusion**

The Alzheimer’s Disease Neuroimaging Initiative (ADNI, <https://adni.loni.usc.edu/>) is a longitudinal, multicenter, observational study launched in 2003. The primary goal of ADNI was to validate biomarkers for mild cognitive impairment (MCI) and early-stage Alzheimer’s disease (AD). This study aims to compare cross-sectional and longitudinal trajectories of diffusion kurtosis imaging along the perivascular space (DKI-ALPS) and diffusion tensor imaging ALPS (DTI-ALPS), and explore their relationships with axonal degeneration, free water, Aβ burden, and cognition. To ensure the accuracy of advanced diffusion modeling techniques (e.g DKI, free water), the inclusion criteria required: (i) MRI data acquired on 3T scanners; (ii) Complete T1-weighted imaging (T1WI) and diffusion-weighted imaging (DWI) datasets; (iii) DWI protocols with b-values of 0, 500, 1000, and 2000 s/mm² and ≥ 50 gradient directions. Exclusion criteria were the following: (i) Age <50 or ≥85 years (*n* = 18); (ii) Missing diagnostic status (*n* = 5); (iii) Poor image quality (*n* = 5). Finally, a total of 211 participants with 362 imaging sessions were included in this study.

**METHOD S2. Aβ-PET processing and SUVR calculation**

Aβ-PET images acquired with ¹⁸F-florbetapir (AV45) or ¹⁸F-florbetaben (FBB) were processed using a standardized pipeline. Briefly, PET images were motion corrected, coregistered to individual T1-weighted MRI, and spatially smoothed. Cortical segmentation was performed using FreeSurfer (version 7.4.1), and cortical regions were defined according to the Desikan–Killiany atlas, covering the entire cerebral cortex. Standardized uptake value ratios (SUVRs) were calculated by normalizing mean cortical tracer uptake to the whole cerebellum reference region.

**METHOD S3. MRI Acquisition**

For all participants, dMRI was acquired using 3T scanners following standardized ADNI protocols with following parameters: b values, 0, 500, 1000, 2000 s/mm^2^; slice thickness, 2 or 2.5 mm; slices spacing, 2 or 2.5 mm; echo time, 71-99 ms; repetition time, 3100-6300 ms; flip angle, 90^0^; matrix size, 116*116 or 128 *128. Full protocol details were available in the ADNI MRI Scanner Protocols (<https://adni.loni.usc.edu/data-samples/adni-data/neuroimaging/mri/mri-scanner-protocols/>).

**METHOD S4. Functional Principal Component Analysis**

Principal component analysis (PCA) is a widely used dimensionality reduction technique that constructs low-dimensional orthogonal components through linear combinations of original variables. As an extension of PCA, Functional PCA (FPCA) employs the Karhunen-Loève expansion to iteratively derive principal components by maximizing projection variance with orthonormal eigenfunctions. FPCA smooths the sparse longitudinal measurements with spaced time points into functional curves, which are decomposed as a mean function plus a sum of products between FPC scores and corresponding eigenfunctions. The number of eigenfunctions is determined by the cumulative explained variance ratio. The mathematical formulation of FPCA can be described as:

$$Y_{i}\left( t \right)= X_{i}\left( t \right)+ \epsilon_{i}=\mu_{i}\left( t \right)+\sum_{i=1}^{\infty} A_{ik}\Phi_{k}\left( t \right)+ \epsilon_{i}$$

Where $Y_{i}\left( t \right)$ and $X_{i}\left( t \right)$ are the observed and true longitudinal dMRI indices at time *t*, $\mu_{i}(t)$ is the smoothed mean function, ${\{A}_{ik}\}{}_{k=1,\ldots,K}$ are the FPC scores, ${\{\Phi}_{k}\left( t \right)\}{}_{k=1,\ldots,k}$ are the corresponding orthonormal eigenfunctions, $\epsilon_{i}$ is a measurement error term, and *K* is the number of FPCs.

**TABLE S1. Comparison of dMRI indices between Groups**

| **dMRI indices** | **Overall** | **HCs** | **CIs** | ***P* Value** | **Cohen’s d** |
| --- | --- | --- | --- | --- | --- |
| **DTI-ALPS** | 1.34 ± 0.23 | 1.37 ± 0.24 | 1.28 ± 0.21 | 0.007* | 0.383 |
| **Left DTI-ALPS** | 1.34 ± 0.26 | 1.38 ± 0.27 | 1.29 ± 0.24 | 0.014* | 0.349 |
| **Right DTI-ALPS** | 1.33 ± 0.23 | 1.37 ± 0.24 | 1.28 ± 0.21 | 0.009* | 0.374 |
| **DKI-ALPS** | 1.53 ± 0.35 | 1.63 ± 0.32 | 1.37 ± 0.34 | <0.001* | 0.770 |
| **Left DKI-ALPS** | 1.54 ± 0.39 | 1.64 ± 0.37 | 1.38 ± 0.36 | <0.001* | 0.722 |
| **Right DKI-ALPS** | 1.51 ± 0.38 | 1.61 ± 0.34 | 1.36 ± 0.38 | <0.001* | 0.674 |
| **PSMD ( × 10^-3^)** | 0.26 ± 0.07 | 0.25 ± 0.08 | 0.26 ± 0.06 | 0.777 | 0.04 |
| **FW-WM** | 0.28 ± 0.03 | 0.28 ± 0.03 | 0.29 ± 0.03 | 0.071 | 0.256 |

Abbreviations: HC = healthy control; CI = cognitive impairment; dMRI = diffusion-weighted magnetic resonance imaging; DTI-ALPS = diffusion tensor imaging analysis along the perivascular; DKI-ALPS = diffusion kurtosis imaging analysis along the perivascular; PSMD = peak width of skeletonized mean diffusivity; FW-WM = free water in white matter.

Continuous variables are presented as means ± SDs, and groups were compared using t-test.

**P* value was <0.05 and considered statistically significant.

**TABLE S2. Comparison of dMRI indices between Groups with GLM Analysis**

| **dMRI indices** | ***P*_model1_ Value** | **Cohen’s d _model1_** | ***P*_model2_ Value** | **Cohen’s d _model2_** |
| --- | --- | --- | --- | --- |
| **DTI-ALPS** | 0.162 | 0.198 | 0.125 | 0.217 |
| **Left DTI-ALPS** | 0.315 | 0.142 | 0.290 | 0.149 |
| **Right DTI-ALPS** | 0.104 | 0.230 | 0.069 | 0.257 |
| **DKI-ALPS** | < 0.001* | 0.659 | < 0.001* | 0.645 |
| **Left DKI-ALPS** | < 0.001* | 0.593 | < 0.001* | 0.587 |
| **Right DKI-ALPS** | < 0.001* | 0.596 | < 0.001* | 0.571 |
| **PSMD ( × 10^-3^)** | 0.922 | 0.013 | - | - |
| **FW-WM** | 0.310 | 0.144 | - | - |

Abbreviations: HC = healthy control; CI = cognitive impairment; dMRI = diffusion-weighted magnetic resonance imaging; DTI-ALPS = diffusion tensor imaging analysis along the perivascular; DKI-ALPS = diffusion kurtosis imaging analysis along the perivascular; PSMD = peak width of skeletonized mean diffusivity; FW-WM = free water in white matter; GLM = general linear model.

Model 1 adjusted for demographic covariates including age, sex, and years of education. Model 2 adjusted for demographic covariates including age, sex, years of education, PSMD, and FW-WM.

**P* value was <0.05 and considered statistically significant.

**TABLE S3. Correlation Analysis Between DTI-ALPS and DKI-ALPS**

| **dMRI indices** | **All Particopants** | **HCs** | **CIs** |
| --- | --- | --- | --- |
| **DTI-ALPS and DKI-ALPS** | r = 0.551; *P* < 0.001* | r = 0.628; *P* < 0.001* | r = 0.370; *P* < 0.001* |
| **Left DTI-ALPS and Left DKI-ALPS** | r = 0.527; *P* < 0.001* | r = 0.614; *P* = 0.004* | r = 0.312; *P* < 0.001* |
| **Right DTI-ALPS and Right DKI-ALPS** | r = 0.469; *P* < 0.001* | r = 0.488; *P* < 0.001* | r = 0.376; *P* = 0.001* |

Abbreviations: HC = healthy control; CI = cognitive impairment; dMRI = diffusion-weighted magnetic resonance imaging; DTI-ALPS = diffusion tensor imaging analysis along the perivascular; DKI-ALPS = diffusion kurtosis imaging analysis along the perivascular.

**P* value was <0.05 and considered statistically significant.

**TABLE S4. Comparison of Trajectory Patterns of dMRI indices between Groups**

| **dMRI indices** | **Cumulative Explained Variance Ratio** | **HCs** | **CIs** | ***P* Value** | **Cohen’s d** |
| --- | --- | --- | --- | --- | --- |
| DTI-ALPS |  |  |  |  |  |
| 1^st^ FPC score | 93.9% | 0.32 ± 1.01 | -0.38 ± 1.03 | < 0.001* | 0.685 |
| 2^st^ FPC score | 99.1% | -0.02 ± 0.29 | 0.02 ± 0.22 | 0.383 | 0.175 |
| DKI-ALPS |  |  |  |  |  |
| 1^st^ FPC score | 87.1% | 0.58 ± 1.44 | -0.69 ± 1.39 | < 0.001* | 0.977 |
| 2^st^ FPC score | 98.0% | 0.03 ± 0.53 | -0.03 ± 0.61 | 0.579 | 0.111 |
| PSMD ( × 10^-3^) |  |  |  |  |  |
| 1^st^ FPC score | 68.1% | -0.06 ± 0.24 | -0.07 ± 0.23 | 0.005* | 0.573 |
| 2^st^ FPC score | 95.5% | -0.01 ± 0.13 | -0.02 ± 0.19 | 0.350 | 0.188 |
| FW Fraction |  |  |  |  |  |
| 1^st^ FPC score | 75.7% | -0.05 ± 0.10 | 0.06 ± 0.13 | < 0.001* | 1.004 |
| 2^st^ FPC score | 96.5% | -0.01 ± 0.06 | 0.01 ± 0.08 | 0.048 | 0.400 |

Abbreviations: HC = healthy control; CI = cognitive impairment; dMRI = diffusion-weighted magnetic resonance imaging; DTI-ALPS = diffusion tensor imaging analysis along the perivascular; DKI-ALPS = diffusion kurtosis imaging analysis along the perivascular; PSMD = peak width of skeletonized mean diffusivity; FW-WM = free water in white matter; FPC = functional principal component.

Continuous variables are presented as means ± SDs, and groups were compared using t-test. The number of FPCs determined using cumulative explained variance ratio thresholded at 95%

**P* value was <0.05 and considered statistically significant.

**TABLE S5. Correlation Analysis Trajectory Patterns of dMRI indices**

| **1^st^ FPC scores of dMRI indices** | **All Particopants** | **HCs** | **CIs** |
| --- | --- | --- | --- |
| **DTI-ALPS and DKI-ALPS** | r = 0.629; *P* < 0.001* | r = 0.685; *P* < 0.001* | r = 0.455; *P* < 0.001* |
| **PSMD and DTI-ALPS** | r = -0.318; *P* = 0.001* | r = -0.135; *P* = 0.327 | r = -0.393; *P* = 0.007* |
| **PSMD and DKI-ALPS** | r = -0.458; *P* < 0.001* | r = -0.369; *P* = 0.006* | r = 0.414; *P* = 0.004* |
| **PSMD and FW-WM** | r = 0.651; *P* < 0.001* | r = 0.596; *P* < 0.001* | r = 0.640; *P* < 0.001* |
| **FW-WM and DTI-ALPS** | r = -0.365; *P* < 0.001* | r = -0.191; *P* = 0.163 | r = -0.326; *P* = 0.027* |
| **FW-WM and DKI-ALPS** | r = -0.545; *P* < 0.001* | r = -0.383; *P* = 0.004* | r = -0.475; *P* < 0.001* |

Abbreviations: HC = healthy control; CI = cognitive impairment; dMRI = diffusion-weighted magnetic resonance imaging; DTI-ALPS = diffusion tensor imaging analysis along the perivascular; DKI-ALPS = diffusion kurtosis imaging analysis along the perivascular; PSMD = peak width of skeletonized mean diffusivity; FW-WM = free water in white matter; FPC = functional principal component.

**P* value was <0.05 and considered statistically significant.

**TABLE S6. Correlation Analysis between dMRI indices and Aβ burden**

| **dMRI indices** | **All Particopants** | **HCs** | **CIs** |
| --- | --- | --- | --- |
| **DTI-ALPS** | r = -0.309; *P* = 0.003* | r = -0.235; *P* = 0.104 | r = -0.143; *P* = 0.379 |
| **DKI-ALPS** | r = -0.468; *P* < 0.001* | r = -0.338; *P* < 0.001* | r = -0.433; *P* < 0.001* |
| **PSMD** | r = 0.113; *P* = 0.157 | r = 0.08; *P* = 0.439 | r = 0.152; *P* = 0.242 |
| **FW-WM** | r = 0.201; *P* = 0.011* | r = 0.037; *P* = 0.720 | r = 0.269; *P* = 0.036* |
| **1^st^ FPC score of DTI-ALPS** | r = -0.277; *P* = 0.009* | r = -0.176; *P* = 0.227 | r = -0.145; *P* = 0.371 |
| **1^st^ FPC score of DKI-ALPS** | r = -0.552; *P* < 0.001* | r = -0.407; *P* = 0.004* | r = -0.478; *P* = 0.002* |
| **1^st^ FPC scores of PSMD** | r = 0.278; *P* = 0.008* | r = 0.187; *P* = 0.197 | r = 0.206; *P* = 0.203 |
| **1^st^ FPC scores of FW-WM** | r = 0.313; *P* = 0.003* | r = 0.078; *P* = 0.592 | r = 0.210; *P* = 0.194 |

Abbreviations: HC = healthy control; CI = cognitive impairment; dMRI = diffusion-weighted magnetic resonance imaging; DTI-ALPS = diffusion tensor imaging analysis along the perivascular; DKI-ALPS = diffusion kurtosis imaging analysis along the perivascular; PSMD = peak width of skeletonized mean diffusivity; FW-WM = free water in white matter; FPC = functional principal component.

**P* value was <0.05 and considered statistically significant.

**TABLE S7. Correlation Analysis between dMRI indices and Cognitive Performance**

| **Characteristics** | **DTI-ALPS** | **DKI-ALPS** | **PSMD** | **FW Fraction** |
| --- | --- | --- | --- | --- |
| **MMSE** | r*_s_* = 0.171; *P_fdr_* = 0.014 | r*_s_* = 0.484; *P_fdr_* < 0.001 | r*_s_* = 0.001; *P_fdr_* = 0.995 | r*_s_* = -0.203; *P_fdr_* = 0.081 |
| **MOCA** | r*_s_* = 0.266; *P_fdr_* < 0.001 | r*_s_* = 0.391; *P_fdr_* < 0.001 | r*_s_* = -0.051; *P_fdr_* = 0.712 | r*_s_* = -0.126; *P_fdr_* = 0.300 |
| **FAQ** | r*_s_* = -0.235; *P_fdr_* = 0.001 | r*_s_* = -0.465; *P_fdr_* < 0.001 | r*_s_* = 0.231; *P_fdr_* = 0.048 | r*_s_* = 0.401; *P_fdr_* < 0; 001 |
| **CDRSB** | r_s_ = -0.291; *P_fdr_* < 0.001 | r_s_ = -0.488; *P_fdr_* < 0.001 | r*_s_* = 0.213; *P_fdr_* = 0.075 | r*_s_* = 0.398; *P_fdr_* < 0.001 |
| **RAVLT** |  |  |  |  |
| **Immediate** | r*_s_* = 0.355; *P_fdr_* < 0.001 | r*_s_* = 0.245; *P_fdr_* = 0.033 | r*_s_* = -0.168; *P_fdr_* = 0.165 | r*_s_* = -0.281; *P_fdr_* = 0.015 |
| **Learning** | r*_s_* = 0.250; *P_fdr_* < 0.001 | r_s_ = 0.227; *P_fdr_* = 0.049 | r*_s_* = -0.074; *P_fdr_* = 0.570 | r*_s_* = -0.197; *P_fdr_* = 0.089 |
| **Forgetting** | r*_s_* = -0.232; *P_fdr_* = 0.001 | r*_s_* = -0.162; *P_fdr_* = 0.165 | r*_s_* = 0.151; *P_fdr_* = 0.218 | r*_s_* = 0.052; *P_fdr_* = 0.682 |
| **Percent forgetting** | r*_s_* = -0.336; *P_fdr_* < 0.001 | r*_s_* = -0.266; *P_fdr_* = 0.021 | r*_s_* = 0.034; *P_fdr_* = 0.791 | r*_s_* = 0.244; *P_fdr_* = 0.035 |
| **ADAS-Cog** |  |  |  |  |
| **ADAS11** | r*_s_* = -0.265; *P_fdr_* < 0.001 | rs = -0.410; *P_fdr_* < 0.001 | r*_s_* = 0.145; *P_fdr_* = 0.231 | r*_s_* = 0.229; *P_fdr_* = 0.049 |
| **ADAS13** | r*_s_* = -0.276; *P_fdr_* < 0.001 | r*_s_* = -0.419; *P_fdr_* < 0.001 | r*_s_* = 0.173; *P_fdr_* = 0.175 | r*_s_* = 0.289; *P_fdr_* = 0.012 |
| **ADASQ4** | r*_s_* = -0.241; *P_fdr_* < 0.001 | r*_s_* = -0.396; *P_fdr_* < 0.001 | r*_s_* = 0.188; *P_fdr_* = 0.118 | r*_s_* = 0.305; *P_fdr_* = 0.008 |
| **LDELTOTAL** | r*_s_* = 0.171; *P_fdr_* = 0.014 | r*_s_* = 0.473; *P_fdr_* < 0.001 | r*_s_* = -0.093; *P_fdr_* = 0.468 | r*_s_* = -0.256; *P_fdr_* = 0.027 |
| **TRABSCOR** | r*_s_* = -0.231; *P_fdr_* = 0.001 | r*_s_* = -0.220; *P_fdr_* = 0.058 | r*_s_* = 0.075; *P_fdr_* = 0.570 | r*_s_* = 0.237; *P_fdr_* = 0.044 |

Abbreviations: HC = healthy control; CI = cognitive impairment; dMRI = diffusion-weighted magnetic resonance imaging; DTI-ALPS = diffusion tensor imaging analysis along the perivascular; DKI-ALPS = diffusion kurtosis imaging analysis along the perivascular; PSMD = peak width of skeletonized mean diffusivity; FW-WM = free water in white matter; MMSE = Mini-Mental State Examination; MoCA = Montreal Cognitive Assessment; FAQ = Functional Activities Questionnaire; CDR-SB = Clinical Dementia Rating Scale Sum of Boxes; RAVLT = Rey Auditory Verbal Learning Test; ADAS-Cog = Alzheimer’s Disease Assessment Scale-Cognitive Subscale; LDELTOTAL = Logical Memory Delayed Recall Total; TRABSCOR = time to complete part B of the Trail Making Test; r*_s_* = Spearman’s rank correlation coefficient; *P_fdr_* = false discovery rate (FDR)-adjusted *P* value.

**P_fdr_* value was < 0.05 and considered statistically significant.

**TABLE S8. Correlation Analysis between Trajectory Patterns and Cognitive Performance**

| **Characteristics** | **1^st^ FPC score of**  **DTI-ALPS** | **1^st^ FPC score of**  **DKI-ALPS** | **1^st^ FPC score of**  **PSMD** | **1^st^ FPC score of**  **FW Fraction** |
| --- | --- | --- | --- | --- |
| **MMSE** | r*_s_* = 0.269; *P_fdr_* = 0.091 | r*_s_* = 0.504; *P_fdr_* < 0.001* | r*_s_* = -0.126; *P_fdr_* = 0.464 | r*_s_* = -0.455; *P_fdr_* = 0.003* |
| **MOCA** | r*_s_* = 0.188; *P_fdr_* = 0.261 | r*_s_* = 0.253; *P_fdr_* = 0.125 | r*_s_* = -0.234; *P_fdr_* = 0.171 | r*_s_* = -0.192; *P_fdr_* = 0.258 |
| **FAQ** | r*_s_* = -0.365; *P_fdr_* = 0.018* | r*_s_* = -0.491; *P_fdr_* = 0.001* | r*_s_* = 0.278; *P_fdr_* = 0.084 | r*_s_* = 0.397; *P_fdr_* = 0.010* |
| **CDRSB** | r_s_ = -0.331; *P_fdr_* = 0.036* | r_s_ = -0.471; *P_fdr_* = 0.002 | r_s_ = 0.374; *P_fdr_* = 0.018* | r_s_ = 0.408; *P_fdr_* = 0.008* |
| **RAVLT** |  |  |  |  |
| **Immediate** | r*_s_* = 0.347; *P_fdr_* = 0.027* | r*_s_* = 0.218; *P_fdr_* = 0.173 | r*_s_* = -0.230; *P_fdr_* = 0.166 | r*_s_* = -0.228; *P_fdr_* = 0.160 |
| **Learning** | r*_s_* = 0.242; *P_fdr_* = 0.130 | r*_s_* = 0.213; *P_fdr_* = 0.182 | r*_s_* = -0.153; *P_fdr_* = 0.378 | r*_s_* = -0.152; *P_fdr_* = 0.359 |
| **Forgetting** | r*_s_* = -0.015; *P_fdr_* = 0.934 | r*_s_* = -0.231; *P_fdr_* = 0.148 | r*_s_* = -0.022; *P_fdr_* = 0.895 | r*_s_* = 0.054; *P_fdr_* = 0.732 |
| **Percent forgetting** | r*_s_* = -0.297; *P_fdr_* = 0.060 | r*_s_* = -0.350; *P_fdr_* = 0.024* | r*_s_* = 0.158; *P_fdr_* = 0.364 | r*_s_* = 0.235; *P_fdr_* = 0.148 |
| **ADAS-Cog** |  |  |  |  |
| **ADAS11** | r*_s_* = -0.420; *P_fdr_* = 0.007* | r*_s_* = -0.413; *P_fdr_* = 0.007* | r*_s_* = 0.123; *P_fdr_* = 0.473 | r*_s_* = 0.215; *P_fdr_* = 0.187 |
| **ADAS13** | r*_s_* = -0.387; *P_fdr_* = 0.014 | r*_s_* = -0.447; *P_fdr_* = 0.004* | r*_s_* = 0.103; *P_fdr_* = 0.536 | r*_s_* = 0.253; *P_fdr_* = 0.116 |
| **ADASQ4** | r*_s_* = -0.224; *P_fdr_* = 0.163 | r*_s_* = -0.485; *P_fdr_* = 0.002* | r*_s_* = 0.052; *P_fdr_* = 0.751 | r*_s_* = 0.172; *P_fdr_* = 0.293 |
| **LDELTOTAL** | r*_s_* = 0.135; *P_fdr_* = 0.410 | r*_s_* = 0.513; *P_fdr_* < 0.001* | r*_s_* = -0.075; *P_fdr_* = 0.644 | r*_s_* = -0.150; *P_fdr_* = 0.361 |
| **TRABSCOR** | r*_s_* = -0.301; *P_fdr_* = 0.060 | r*_s_* = -0.159; *P_fdr_* = 0.333 | r*_s_* = 0.142; *P_fdr_* = 0.411 | r*_s_* = 0.384; *P_fdr_* = 0.015* |

Abbreviations: HC = healthy control; CI = cognitive impairment; dMRI = diffusion-weighted magnetic resonance imaging; DTI-ALPS = diffusion tensor imaging analysis along the perivascular; DKI-ALPS = diffusion kurtosis imaging analysis along he perivascular; PSMD = peak width of skeletonized mean diffusivity; FW-WM = free water in white matter; MMSE = Mini-Mental State Examination; MoCA = Montreal Cognitive Assessment; FAQ = Functional Activities Questionnaire; CDR-SB = Clinical Dementia Rating Scale Sum of Boxes; RAVLT = Rey Auditory Verbal Learning Test; ADAS-Cog = Alzheimer’s Disease Assessment Scale-Cognitive Subscale; LDELTOTAL = Logical Memory Delayed Recall Total; TRABSCOR = time to complete part B of the Trail Making Test; FPC = functional principal component; r*_s_* = Spearman’s rank correlation coefficient; *P_fdr_* = false discovery rate (FDR)-adjusted *P* value.

**P_fdr_* value was < 0.05 and considered statistically significant.
